# Research on Structural Optimization of High-Sensitivity Torque Sensors for Robotic Joints

**DOI:** 10.3390/s26020649

**Published:** 2026-01-18

**Authors:** Yizhou Chen, Shenglin Yu, Jinjie Xu

**Affiliations:** 1 School of Electronic and Information Engineering, Nanjing University of Information Science and Technology, Nanjing 210044, China; 202312490143@nuist.edu.cn (Y.C.); 202312490177@nuist.edu.cn (J.X.); 2 School of Integrated Circuits, Nanjing University of Information Science and Technology, Nanjing 210044, China

**Keywords:** humanoid robot, collision perception, torque sensor, response surface optimization, sensitivity

## Abstract

To address the urgent need for real-time and high-precision torque perception in robotic manipulators operating in complex environments, this study focuses on the structural optimization design of joint torque sensors. By proposing a novel hourglass-hole spoke-type elastic body structure, a systematic parametric optimization study was conducted with the objectives of improving material utilization and output sensitivity. To enhance optimization efficiency, single-factor experiments and explanatory notes on parameter selection ranges were incorporated to identify factors significantly influencing the target response and to determine their appropriate experimental ranges. Building upon this, the Box–Behnken experimental design method was employed, combined with response surface methodology, to perform multi-objective optimization on the key dimensions of the elastic body. Experimental results demonstrate that the optimized sensor structure achieved a 13.1% improvement in material utilization and an 11.9% increase in sensitivity. The baseline sensitivity of the final sensor reached 0.558 mV/N·m, representing a 19.2% enhancement compared to the optimized dumbbell-hole structure, while material utilization was also improved by 3.1%. This study proposes a novel high-sensitivity hourglass-hole spoke-type elastic body configuration and establishes an efficient response surface optimization framework applicable to the structural design of joint torque sensors fabricated from linear elastic materials, offering new insights for the design and optimization of high-sensitivity torque sensors.

## 1. Introduction

Driven by global market demand, robots have found extensive applications across diverse sectors such as industry, healthcare, and service domains [1]. With continuous technological advancement, mobility, functionality, and human–machine interaction have become central topics of interest within the robotics field [2]. Among various robotic forms, humanoid robots, owing to their anthropomorphic appearance and motion patterns, demonstrate distinctive advantages in collaborative scenarios [3]. However, the rapid development of robots also raises critical safety concerns [4]. In human–robot collaborative environments, the inability of robots to adapt their behavior in complex or unexpected situations may result in unintended collisions [5]. Such incidents not only risk damaging the robots and causing economic losses but may also pose potential hazards to the surrounding environment and nearby personnel [6]. Furthermore, the integration of collision perception capabilities contributes to mitigating the risk of collisions occurring during the motion of robotic arms. Nevertheless, in industrial and other complex environments, uncertainties such as dust, oil contamination, unstable lighting conditions, and object occlusions can substantially compromise the reliability of existing collision detection methods [7].

Existing collision perception approaches can generally be classified into non-contact and contact-based methods. In the non-contact category, vision-based obstacle detection systems [8] rely heavily on camera resolution, computational power, and environmental conditions. Under challenging scenarios such as dusty or poorly lit environments, or when obstacles obstruct the field of view, image features become difficult to extract, thereby preventing robots from accurately identifying collisions [9]. Similarly, radar-based systems may suffer from signal reflections that produce multipath effects, which in turn compromise detection reliability [10]. Path-planning strategies, on the other hand, are unable to reliably predict collisions in unknown or dynamic environments, making them unsuitable for humanoid robots that must maintain continuous motion [11]. For contact-based approaches, current monitoring methods detect collisions by observing abnormal motor current fluctuations. However, since they measure motor input rather than output torque, nonlinear errors from reducers or other transmission components reduce detection accuracy [12]. Tactile sensors, which are typically distributed across the robot’s surface, are also prone to performance degradation; accumulated dust or contamination can impair their sensitivity and precision, thereby diminishing collision sensing reliability [13,14].

As a core component enabling force sensing and control in robotic joints, torque sensors can directly measure the force values generated by external collisions or load variations through real-time monitoring of joint output torque. Compared to other sensing methods, joint torque sensors offer several distinct advantages. Firstly, they are typically installed internally within the joint, making them immune to environmental factors such as lighting conditions, dust, oil contamination, and occlusions, thus ensuring reliable operation in complex application scenarios. Secondly, one of the key bottlenecks in the commercialization of robotics is “cost control”. Torque sensors with simple structural designs can reduce costs throughout the entire lifecycle, with lower integration barriers, reduced manufacturing and maintenance expenses, thereby facilitating the commercialization of humanoid robots. Furthermore, a complete robotic system often adopts a strategy of fusing multiple sensing modalities [15]. The design objective of joint torque sensors is to achieve coexistence with other sensing systems rather than to replace them. They serve as an indispensable, last line of safety defense, ensuring robotic reliability even when other sensing systems fail due to complex external factors.

More importantly, joint torque sensors are not only capable of enabling collision detection but also support applications such as compliant control and safe human-robot interaction, serving as a critical foundational element for enhancing both the safety and intelligence of robotic systems. Therefore, research centered on torque sensors for robotic joints, particularly in the areas of structural design and performance optimization, holds significant importance for improving a robot’s perception capabilities and operational safety in complex environments. This underscores the persistently unique and crucial technological value of torque sensors in robotic applications.

Torque sensors are primarily categorized into optical, capacitive, and resistive strain-gauge types. Considering the requirements for integration into robotic joints—such as structural simplicity, reliability, compact thickness, and fast response—the resistive strain-gauge torque sensor was selected for detailed investigation. Regarding resistive strain-gauge sensors, scholars have achieved a series of significant advancements [16]. Lou et al. [17] proposed a dumbbell-hole spoke-structured joint torque sensor for robotic collision detection, which improved sensitivity and stress uniformity, achieving a sensitivity of up to 33.49 mV/N·m. Han et al. [18] designed a novel floating-beam and support-beam miniature joint torque sensor with a sensitivity of 2.48 mV/V for measuring finger joint torque. To further increase the average strain on the sensing surface of the elastic body based on the dumbbell-hole structure, Nguyen et al. [19] introduced a novel inclined sensing surface design for the spokes, ultimately achieving a sensitivity of 1.65 mV/N·m. Tian et al. [20] developed an ultra-compact double-hole support structure for joint torque sensors, enabling testing of three sensors with different resolutions while maintaining appropriate stiffness. To ensure sensor reliability, Mohandas et al. [21] proposed a pear-shaped hole torque sensor design based on high-sensitivity strain gauges, offering a sensitivity of 38.67 mV/N·m and reducing the risk of mechanical resonance. Kim et al. [22] investigated other sensor performance aspects and proposed a tapered-lock spoke-type sensor with high torsional stiffness and effective crosstalk suppression, achieving a sensitivity of 17.48 mV/N·m.

Previous research on joint torque sensors indicates that sensitivity is a critical performance metric that directly influences sensor accuracy. In torque sensors based on the strain-gauge measurement principle, the applied torque first induces elastic deformation in the elastic body, generating corresponding strain in the area where the strain gauges are bonded. The resistance of the strain gauges then changes with the strain, and this change is converted into a weak differential voltage signal through a Wheatstone bridge, which is finally amplified and output by the signal conditioning circuit. Therefore, sensitivity performance should not be evaluated solely based on the final output; rather, it is the combined result of the strain response capability at the strain-gauge bonding area of the elastic body and the amplification capability of the circuit.

Since the gain selection of the signal conditioning circuit primarily serves to match the signal amplitude with the ADC’s dynamic range, it has a clear engineering limit in enhancing sensor sensitivity performance. In contrast, improving strain response capability through structural optimization can fundamentally increase sensor sensitivity. Thus, research on optimizing the strain characteristics of the elastic body structure is more conducive to revealing the underlying principles of sensitivity enhancement in torque sensors. Material utilization is typically defined as the ratio of the maximum stress under the rated maximum working load to the material’s yield strength. This metric reflects the extent to which the material’s load-bearing capacity is utilized under the sensor’s extreme operating conditions and often correlates positively with sensitivity.

Moreover, in existing torque sensors used for joint torque perception, performance often suffers from insufficient sensitivity and material utilization. When applying response surface methodology (RSM) alone to optimize torque sensor structures, identifying significant factors and defining their ranges typically requires extensive RSM experimental designs and iterative screening, significantly increasing the complexity of the optimization process.

To address these issues, this study designs a high-sensitivity hourglass-hole spoke-type joint torque sensor through material performance comparison and structural selection. Additionally, a comprehensive systematic optimization method based on RSM is proposed. Prior to RSM optimization, single-factor experiments for structural optimization and explanatory notes on parameter range selection are introduced. This approach screens for factors significantly influencing the target response and determines reasonable experimental ranges, reducing the need for experimental redesign due to inappropriate parameter selections and ranges. Consequently, it accelerates the identification of potential optimal solutions for the structural model, enhances optimization efficiency, and improves the rigor of the entire optimization process. This significantly increases the reliability and engineering interpretability of the optimization results, offering general applicability for the structural optimization of joint torque sensors made from linear elastic materials. The final optimized design improves the sensor’s material utilization and sensitivity, ensures uniform and concentrated strain distribution on the sensing surface, reduces sensor thickness for enhanced lightweight characteristics, and thereby enables faster and more accurate perception of joint torque in robots.

## 2. Principle of Sensors

**Figure 1:**
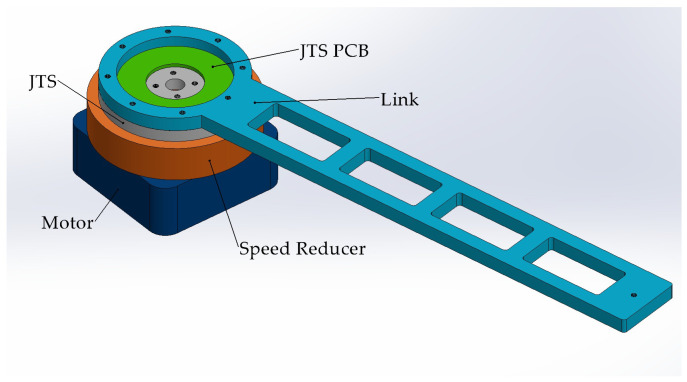
Internal composition of the robotic joint.

The torque sensor investigated in this study is integrated within the robotic arm joint. As illustrated in Figure 1, the joint primarily consists of a motor, speed reducer, joint torque sensor (JTS), JTS PCB, and the link.

The components are connected via bolt holes and bolts. Specifically, the inner flange of the joint torque sensor is equipped with four threaded holes and is bolted to the speed reducer, while the outer flange features eight threaded holes and is bolted to the connecting arm. During static calibration, the fixed end of the base is secured to an optical precision vibration isolation platform using bolts, replacing the motor and speed reducer. This setup constrains and fixes the inner flange of the sensor via bolt connections. Meanwhile, the force applied to the link drives the link to generate a torque load, which is subsequently transferred to the sensor’s outer flange connected to it.

**Figure 2:**
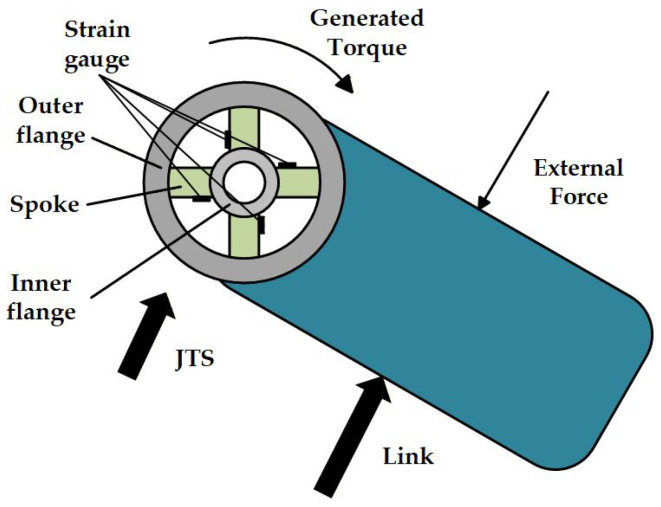
Schematic diagram of the Torque generation process.

When an external force acts on the link, it generates an external torque on the outer flange of the joint torque sensor connected to it. The direction of this torque is opposite to that of the internal driving torque produced by the robot’s own motor, as it resists the joint motion. The total torque at the joint comprises both the internal motor driving torque and the torque generated by the external force. The elastic body’s outer flange, which connects the link, is subjected to this torque while the inner flange remains fixed, causing the spokes to twist. Strain gauges affixed to the spoke surfaces deform correspondingly, inducing changes in the resistance of the metallic wires embedded in the gauges. This establishes a functional relationship between the applied load and the resistance variation. A Wheatstone bridge converts these resistance changes into measurable voltage variations, which are then amplified to obtain a voltage signal. Consequently, a functional relationship between the external load and the sensor’s voltage output is established. A schematic diagram illustrating the specific process of torque generation from an external force is shown in Figure 2.

The sensitivity of a strain gauge to mechanical strain, commonly expressed by the gauge factor *GF*, is defined as the ratio of the relative change in resistance ΔR/R to the applied strain ε:

(1) $$GF=\frac{\Delta R/R}{\varepsilon }$$

In the sensor design, four strain gauges are affixed to the lateral surfaces of the spokes on the elastic wheel structure. Since the high-strain region on the spokes is relatively limited, efficient utilization of this region is essential. To achieve this, strain gauges with a substrate size of 6.8 mm × 4.2 mm and a grid size of 3 mm × 3 mm are employed. Accordingly, a 3 mm × 3 mm rectangular area at the center of the high-strain region on each spoke surface is designated as the bonding location for the strain gauge grids.

**Figure 3:**
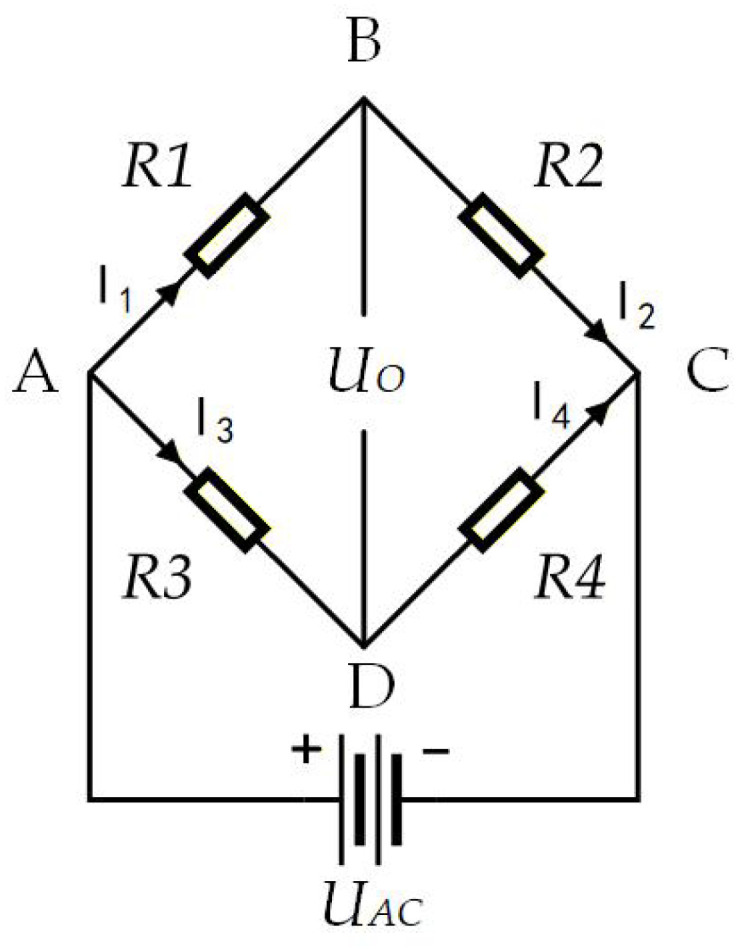
Schematic diagram of the Wheatstone bridge.

When the sensor is subjected to an external torque load, the resulting deformation causes changes in the strain gauge resistance. These resistance variations are then converted into voltage changes via a Wheatstone bridge, as illustrated in Figure 3.

Based on Equation (1), the output voltage can be approximated as:

(2) $$U_{0}\approx \frac{U_{AC}}{4}GF\left(\varepsilon_{1}-\varepsilon_{2}-\varepsilon_{3}+\varepsilon_{4}\right)$$

## 3. Structural Design

In this section, the requirements for torque measurement in robotic collision detection are first discussed, followed by the corresponding sensor design specifications.

### 3.1. Design Objectives

The primary objective of this study is to propose and validate the advantages of a novel hourglass-hole spoke-type sensor structure in enhancing sensitivity. A widely accepted collaborative robot joint dimension from both academia and industry is adopted as a representative design case. This reference dimension is primarily derived from the dumbbell-hole torque sensor proposed by Lou et al. [17], ensuring the typicality and rationality of the design case.

The outer flange is configured with an outer diameter of 78 mm and an inner diameter of 60 mm, while the inner flange is designed with an outer diameter of 30 mm and an inner diameter of 10 mm. To ensure structural compactness and lightweight characteristics, the initial thickness is specified as 7 mm. The robotic arm typically has a length of 30 cm, meaning the maximum force arm length is 300 mm. Considering safety in this study, the maximum load at the end-effector is set at 10 kg, leading to a torque sensor range of 30 N·m. To ensure safety and prolong the service life of the elastic body, the overload capability is set at 250%, meaning that when a torque of 75 N·m is applied, the maximum stress in the elastic body must not exceed its yield strength. However, in practical scenarios, external forces may not always act at the very end of the force arm. Assuming the external force is applied at a position 10 mm away from the sensor, the minimum resolution of the sensor should be set at 0.1 N·m. The sensor is required to operate within the elastic range of the elastic body, and its output voltage should exhibit a linear relationship with the external torque. By taking the initial time as zero with 0 V output, the sensor sensitivity S can be expressed as follows:

(3) $$\begin{array}{cc} S & =\frac{\Delta U_{0}}{\Delta M_{y}}\\ & =\frac{U_{0}-0}{M_{y}-0}\\ & =\frac{U_{AC}GF\left(\varepsilon_{1}-\varepsilon_{2}-\varepsilon_{3}+\varepsilon_{4}\right)}{4M_{y}} \end{array}$$

Here, My represents the torque applied along the y-axis, and the input voltage $U_{AC}$ is 5 V. The strain gauge gauge factor GF is 2, and under ideal conditions, the strain values at all four positions are identical. For comparison, the dumbbell-hole spoke-type torque sensor designed by Lou et al. [17] exhibits an average strain of $3510\times 10^{-6}$ at the strain gauge locations when subjected to a torque of 75 N·m. According to Equation (5), the corresponding sensitivity S of the dumbbell-hole spoke torque sensor is 0.468 mV/N·m. Therefore, the designed sensor must achieve a sensitivity exceeding that of the dumbbell-hole design to meet the target requirements. The specific design specifications are listed in Table 1.

### 3.2. Design Scheme

#### 3.2.1. Structural Type Selection

Strain gauge-based joint torque sensors can be categorized according to the operating mode of the strain gauges, primarily into shear-strain gauge types and bending-strain gauge types. Among them, the shear-strain gauge joint torque sensors commonly adopt structures such as the disk type and the double-layer ring type. When the fixed flange is secured, applying a torque load to the loading flange induces torsion in the spokes. In shear-strain gauge sensors, the strain gauges are affixed to the front surface of the spokes, which simplifies the bonding process. These gauges measure the shear strain on the spoke surface, resulting in a relatively uniform and non-concentrated stress-strain distribution. Consequently, the average strain at the strain gauge locations is relatively small, leading to lower sensor sensitivity. However, due to the absence of stress concentration, this structural design typically offers a longer service life.

**Figure 4:**
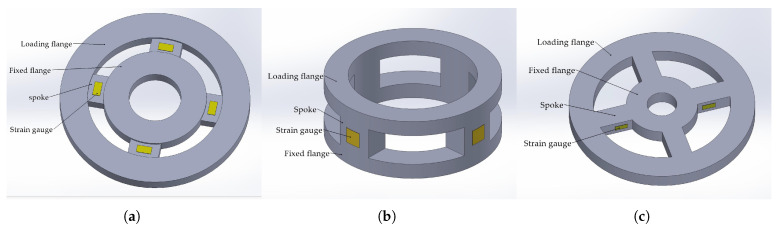
Classification of Elastic Body Structures: (**a**) Disk type. (**b**) Double-layer ring type. (**c**) Spoke-wheel type.

In contrast, bending-strain gauge joint torque sensors are typically based on a spoke-wheel structure, with strain gauges bonded to the sides of the spokes. Although the bonding process is comparatively more challenging, bending strain tends to be more pronounced than shear strain, and the stress-strain distribution is more concentrated. This makes such structures suitable for sensor designs requiring high sensitivity. Since the objective of this study is to enhance sensitivity, the spoke-wheel structure has been selected. The three structural configurations are illustrated in the Figure 4.

**Figure 5:**
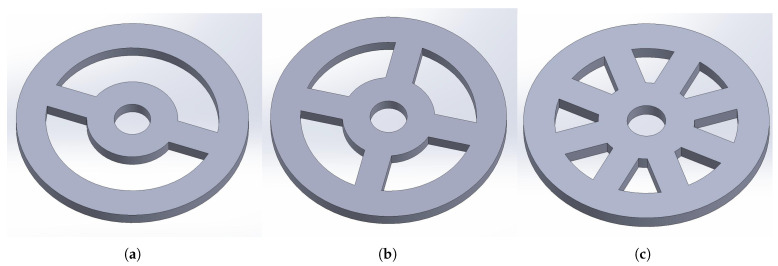
Classification of Spoke-Wheel Structures: (**a**) Double-spoke type. (**b**) Cross-spoke type. (**c**) Double-cross-spoke type.

Spoke-wheel torque sensors can be further categorized based on the number of spokes into double-spoke, cross-spoke, and double-cross-spoke structures. The double-spoke structure features only two symmetrical spokes, meaning that the stress generated by external forces is solely borne by these two spokes. The resulting concentration of load leads to significant deformation of the spokes, yielding high sensitivity. However, this design exhibits weaker resistance to failure, characterized by lower stiffness and load-bearing capacity. In contrast, the double-cross-spoke structure incorporates a greater number of spokes, distributing the external force across more load-bearing elements. Furthermore, the dense arrangement of the spokes substantially disperses the applied load, resulting in higher structural stiffness and load-bearing capacity. Yet, this configuration experiences smaller deformation and consequently lower sensitivity. Additionally, since the strain gauges are affixed to the sides of the spokes, an increase in the number of spokes complicates the bonding process. The joint torque sensor in this study must ensure a certain load-bearing capacity, achieve relatively high sensitivity, and maintain a relatively straightforward strain gauge bonding process. Therefore, the cross-spoke structure, which offers balanced performance, has been selected. The three structural configurations are illustrated in the Figure 5.

The spoke can be modeled as a cantilever beam in engineering mechanics. Based on the bending stress formula for beams and the proportional relationship between stress and strain for linearly elastic materials, the bending strain formula for the spoke is given as follows:

(4) $$\varepsilon =\frac{My}{EI}$$

where *M* represents the bending moment of the beam cross-section, reflecting the effect of external forces on the bending of the beam; *y* denotes the distance from the calculation point to the neutral axis, with the neutral axis being the line within the beam cross-section where stress is zero—typically the symmetry axis for beams with symmetric cross-sections; *E* is the Young’s modulus of the material; and *I* is the moment of inertia of the cross-section about the neutral axis, indicating the section’s resistance to bending. A larger value of *I* corresponds to greater bending resistance, resulting in smaller generated strain.

When a beam is subjected to a moment load at one end, according to the formula, stress and strain are highly concentrated at the location farthest from the neutral axis, which is the root of the fixed end. Such concentrated strain makes it challenging for strain gauges to adequately cover and measure the strain. When a through-hole is added at the center of the beam, under an unchanged bending moment, the bending resistance of the cross-section at the location of the through-hole decreases, i.e., the moment of inertia of the cross-section about the neutral axis near the through-hole is reduced. Consequently, the strain at that location becomes more concentrated and increases, thereby transferring part of the stress and strain from the beam root to the region near the through-hole at the beam root. This explains why, when a through-hole is added, strain becomes more concentrated near the center of the spoke, making it easier for strain gauges to be attached and measure the regions of higher structural strain.

#### 3.2.2. Structural Model and Parameters

Based on the through-hole design of the conventional wheel-spoke sensor, this study proposes a novel hourglass-shaped through-hole wheel-spoke elastic body design. This approach aims to enhance sensitivity while maintaining uniform stress distribution.

**Figure 6:**
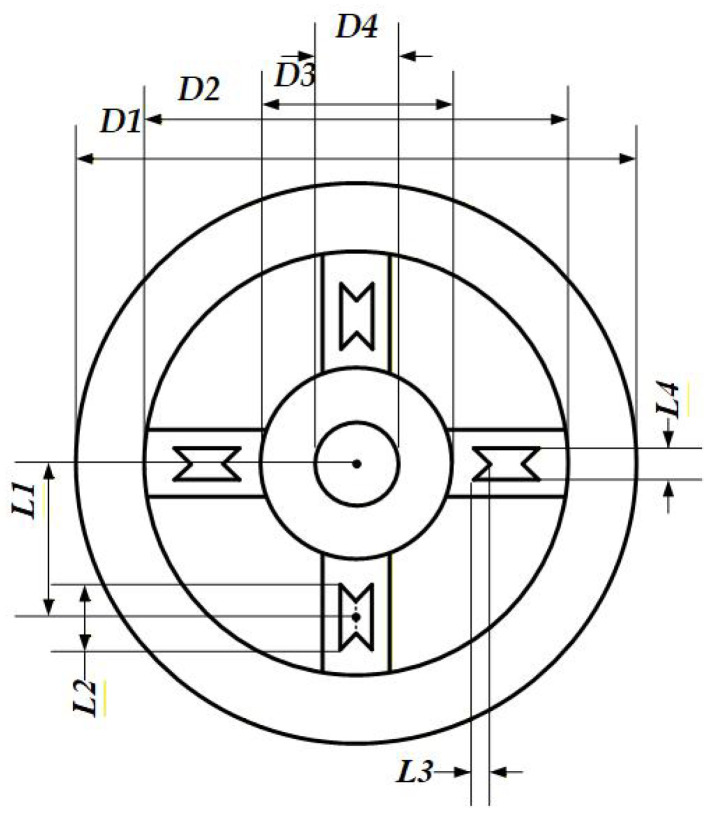
Elastic structure.

The structure of the sensor’s elastic element is illustrated in Figure 6, where the spoke width is 8 mm and the elastic element thickness *H* is 7 mm.

The specific dimensional values are presented in Table 2. For the initial design, the maximum stress of the sensor is 441 MPa, and the average strain at the strain gauge bonding locations is $3740\times 10^{-6}$.

#### 3.2.3. Selection of Elastic Body Material

The material properties of the elastomer significantly influence both the structural dimensions and mechanical characteristics of the sensor. Therefore, selecting an appropriate material based on the sensor’s design specifications is essential. For joint torque sensors in robots, the following factors must be comprehensively considered:

First, the material should exhibit a relatively low Young’s modulus. This property directly reflects the material’s stiffness and determines its resistance to deformation. Within the elastic deformation range, under identical loading conditions, a higher Young’s modulus results in greater resistance to deformation and higher stiffness, but at the cost of reduced sensitivity. Since the sensor in this study requires high sensitivity while maintaining sufficient stiffness, an elastomer material with a low Young’s modulus is preferred.

Second, the material should have a low density. As the sensor is to be integrated into the joints of a robotic arm, lightweight design is critical. A lower material density contributes to reducing the overall weight and power consumption of the robotic arm.

Additionally, the material must demonstrate good machinability. Machinability refers to the ease and stability of the material during mechanical processing. Strain gauge sensors measure strain to infer force information, necessitating high machining precision for the elastomer. If the material is difficult to machine or results in uneven surfaces, strain gauge adhesion may be compromised or adhesive layer thickness may become inconsistent, leading to output deviations.

Finally, the material should possess relatively high strength. The operational principle of strain-based torque sensors requires that measurements remain within the elastic deformation range. According to the design specifications, the maximum stress induced in the elastomer under the maximum overload condition must not exceed the yield strength. Exceeding this limit causes plastic deformation, resulting in permanent, irreversible changes. This leads to nonlinear strain gauge outputs, zero-point drift even after unloading, and degraded sensitivity after repeated loading. If the applied load significantly surpasses both the yield strength and ultimate tensile strength, elastomer failure may occur, rendering the sensor inoperable.

Considering these factors comprehensively, commonly used materials for torque sensor elastomers include 7075-T6 aluminum alloy, 6061-T6 aluminum alloy, 2A12-T4 aluminum alloy, 17-4PH stainless steel, Ti-6Al-4V titanium alloy, and 42CrMo steel. The Table 3 below summarizes the fundamental properties of these materials, along with the maximum stress in the elastomer model and the average strain at the strain gauge location under the 250% overload condition of 75 N·m.

As evidenced by the data presented in the Table 3, while 6061-T6 and 2A12-T4 aluminum alloys exhibit relatively low Young’s modulus, low density, and good machinability, their respective yield strengths are both exceeded by the maximum stress generated under the 75 N·m overload condition. This indicates that these materials would undergo plastic deformation under extreme loading, rendering them unsuitable for the elastomer application in this study. The Ti-6Al-4V titanium alloy, despite its high strength characteristics, demonstrates significantly higher density and Young’s modulus compared to aluminum alloys. These properties would compromise both the sensitivity and lightweight design objectives of the sensor. Furthermore, its poor machinability and associated high manufacturing costs present substantial practical limitations for implementation in humanoid robot joint torque sensors. Both 17-4PH stainless steel and 42CrMo steel exhibit excessive density and elevated Young’s modulus values, which would adversely affect the sensor’s lightweight design and sensitivity performance. Through comprehensive comparative analysis, 7075-T6 aluminum alloy emerges as the optimal choice for the sensor elastomer material. It maintains a favorable balance of low Young’s modulus and density, combined with excellent machinability. Crucially, under the specified overload condition, the maximum stress remains safely below the material’s yield strength, ensuring structural integrity while meeting all design requirements. Therefore, 7075-T6 aluminum alloy has been selected as the material for the torque sensor elastomer in this research.

#### 3.2.4. Structural Comparison

**Figure 7:**
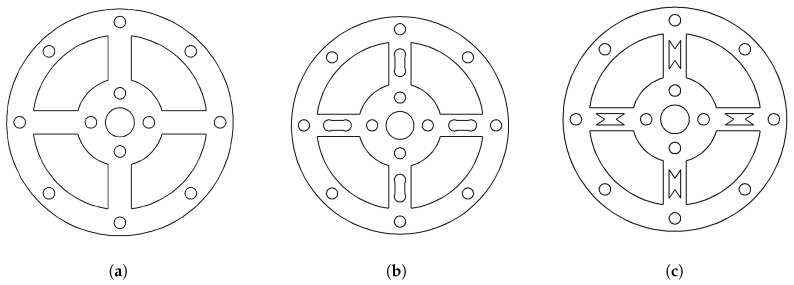
Structural Model Comparison: (**a**) Solid Spoke Configuration. (**b**) Dumbbell-hole Configuration. (**c**) Hourglass-hole Configuration.

**Figure 8:**
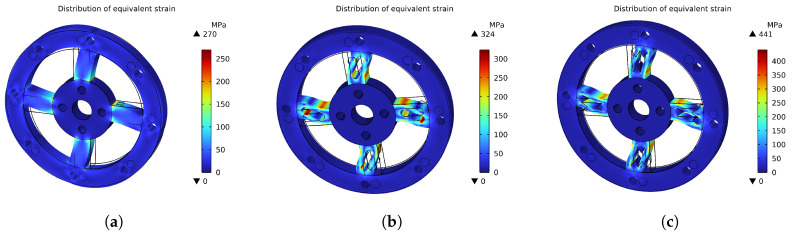
Stress Distribution Comparison: (**a**) Stress Distribution in Solid Spoke Structure. (**b**) Stress Distribution in Dumbbell-hole Structure. (**c**) Stress Distribution in Hourglass-hole Structure.

**Figure 9:**
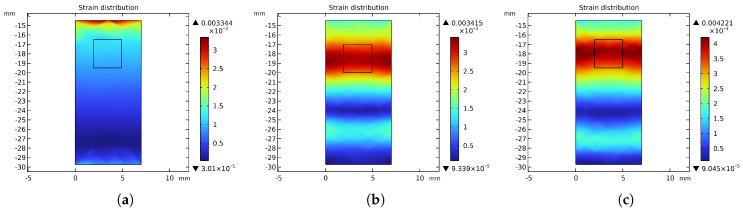
Strain Distribution Comparison: (**a**) Strain Distribution in Solid Spoke Structure. (**b**) Strain Distribution in Dumbbell−hole Spoke Structure. (**c**) Strain Distribution in Hourglass−hole Spoke Structure.

This section presents a comparative analysis of the structure and performance of three initial-dimension-based joint torque sensor designs: the solid type, the dumbbell-hole type, and the hourglass-hole type. Figure 7 illustrates the structural models of the three torque sensor configurations. Figure 8 compares the maximum stress distributions across the three models. Figure 9 displays the strain distribution at the strain gauge attachment area on the spokes for each model. Table 4 provides a comparative summary of the stress-strain distribution and key performance metrics for all three sensor models, with a uniform thickness of 7 mm.

As shown in Figure 9, the strain in the solid sensor is predominantly concentrated on one side of the spokes, complicating subsequent strain gauge installation and hindering effective torque signal acquisition. In contrast, the spoked structures with through-holes demonstrate more uniform strain distribution, with high-strain regions shifted toward the central area of the spokes, thereby facilitating strain gauge attachment. The 3 mm × 3 mm rectangular area indicates the mounting position of the strain gauge grid. Comparative analysis further reveals that the hourglass-hole torque sensor exhibits superior performance in certain aspects, showing significant improvements in both material utilization and sensitivity compared to the other structural configurations.

## 4. Structural Optimization

### 4.1. Optimization Model

For structural optimization, sensor sensitivity is the primary criterion. The goal is to maximize sensitivity while ensuring that the maximum stress does not exceed the material’s yield strength.

From Equation (5), it is evident that under the same applied torque, a larger strain at the strain gauge bonding locations corresponds to higher sensor sensitivity. Therefore, the average equivalent strain $\varepsilon_{t}$ at the strain gauge bonding area can be defined as the objective function for optimization:

(5) $$f\left(x\right)=\varepsilon_{t}$$

In addition to enhancing sensitivity, the torque sensor must maintain adequate strength to ensure operational safety. According to the fourth strength theory, the maximum stress of the elastic element should not exceed the material’s yield strength, expressed as:

(6) $$g\left(x\right)=\sigma_{\max}-{\left[\sigma \right]}_{yield}\leq 0$$

In practical applications, to ensure that the structure does not undergo plastic deformation under applied loads while maximizing material utilization, the average equivalent stress of the structure should not only remain below the material’s yield strength but also approach it. Therefore, the constraint can be expressed as:

(7) $$n=\frac{\sigma_{\max}}{{\left[\sigma \right]}_{yield}}\to 1$$

Here, $\sigma_{\max}$ denotes the maximum stress in the elastic body, and ${\left[\sigma \right]}_{yield}$ represents the yield strength of the selected 7075-T6 aluminum alloy.

Due to the dimensional constraints of the humanoid robot’s arm joints and motors, *D*1, *D*2, *D*3, *D*4 and the spoke width are fixed and cannot be used as optimization variables. Therefore, *L*1, *L*2, *L*3, *L*4 and the elastic body thickness *H* were initially selected as variable parameters for five-level single-factor experiments to identify the most significant factors, thereby reducing the complexity of the subsequent response surface experiments. The experimental models were created in SolidWorks 2022 and imported into COMSOL Multiphysics 6.2 for finite element analysis to obtain the average strain f(x) at the strain gauge locations and the maximum stress g(x) of the elastic body. In the single-factor experiments, all other factors were held constant while only the target factor was varied, isolating its influence on the results.

**Figure 10:**
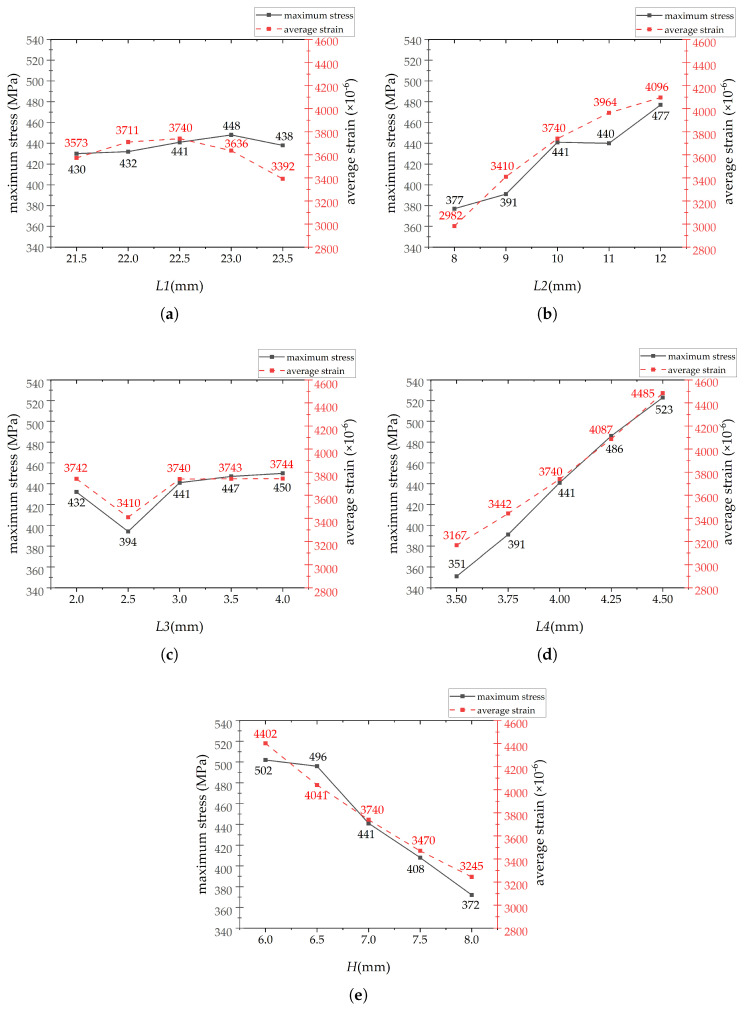
Single−factor experiment on the influence of different parameters on maximum strain and average stress: (**a**) *L*1’s single−factor experiment. (**b**) *L*2’s single−factor experiment. (**c**) *L*3’s single−factor experiment. (**d**) *L*4’s single−factor experiment. (**e**) *H*’s single−factor experiment.

Figure 10 illustrates the influence of parameters *L*1, *L*2, *L*3, *L*4 and the elastomer thickness *H* on both the average strain at the strain gauge location and the maximum stress within the elastomer. Given the constraints imposed by the elastomer dimensions, strength requirements, and optimization objectives, it is necessary to define the parameter ranges for the single-factor experiments. The specific determination process is as follows:

For *L*1, given the dimensional limitations of the robotic arm joint, the spoke length between the inner and outer flanges was designed as 15 mm. When *L*1 is smaller than 21.5 mm or larger than 23.5 mm, and *L*2 is assigned a value greater than 10 mm, the through-hole structure becomes excessively close to the edge of the spoke, leading to improper stress and strain distribution. Moreover, when *L*1 falls outside the range of 21.5 mm to 23.5 mm, the variations in stress and strain remain minimal, indicating that it is a non-significant factor. Therefore, the range of 21.5 mm to 23.5 mm was selected. When *L*2 is set to 13 mm, the maximum stress of the elastomer far exceeds the yield strength of 503 MPa, and the through-hole structure again becomes too close to the spoke edge. If *L*2 is set below 8 mm, the average strain becomes too small. Since the objective is to maximize the average strain, values below 8 mm were excluded from the range. Similar to *L*1, *L*3 exhibits minimal variation in stress and strain and is considered a non-significant factor. Additionally, when *L*3 exceeds 4 mm and *L*2 is set below 10 mm, the shape of the through-hole becomes distorted, which is undesirable. Thus, such values were excluded from the range. When *L*4 and *H* are set to 4.5 mm and 6 mm, respectively, the stress approaches or exceeds the yield strength. Further increasing these values is not meaningful. Moreover, the stress-strain curves for both parameters are monotonic. Therefore, the range was selected to prioritize larger average strain values, as selecting smaller values would not contribute to the objective.

As can be observed from the Figure 10, *L*1 and *L*3 exhibit negligible influence on the average strain, whereas *L*2, *L*4 and H demonstrate significant effects on the sensor’s average strain. Therefore, *L*2, *L*4 and *H* are selected as the parameter variables for the response surface optimization experiments.

A subsequent three-factor, three-level response surface experiment is required. Therefore, three consecutive values must be selected from the five initial values within the ranges of *L*2, *L*4 and *H* to represent the low, medium, and high levels for each factor.

First, as explained in the text, a larger average strain corresponds to higher sensor sensitivity. To achieve the objective of high sensitivity, the selected range for optimization should consist of three consecutive values associated with larger average strains. Second, to ensure the sensor’s strength does not fail, the maximum stress for the selected factors during optimization must not exceed the yield strength of 503 MPa. Moreover, the optimal solution should exhibit a maximum stress close to the constraint boundary. To ensure the three-level range encompasses potential optimal solutions, the selected range should feature both high maximum stress and high average strain, while being proximate to the constraint boundary. Finally, the differences in maximum stress and average strain across the three levels should be sufficiently distinct. Insufficient distinction may cause minor variations in the factor to be obscured by numerical noise. During ANOVA, this would result in a large *p*-value for the factor, leading the software to deem the factor insignificant and thus fail to identify its true influence. It would also weaken the model’s predictive ability, resulting in a very flat response surface and unreliable optimization outcomes.

Based on the above requirements, for *L*2, the interval from 10 mm to 11 mm is not selected as the three-level range because the variation in maximum stress within this interval is not significant. Therefore, the range from 8 mm to 10 mm is preliminarily chosen as the parameter interval. For *L*4, since the maximum stress at 4.5 mm exceeds the yield strength of 503 MPa, this value is excluded from the three-level range. Instead, the interval from 3.75 mm to 4.25 mm, which has the greatest influence on both maximum stress and average strain among the remaining values, is selected as the parameter interval. For *H*, the interval from 6 mm to 6.5 mm is excluded from the three-level range because the variation in maximum stress within this interval is not significant, and the maximum stress at 6 mm reaches 502 MPa, approaching the yield strength of the elastomer. To ensure sensor safety, this interval is omitted. Instead, the range from 6.5 mm to 7.5 mm, which has the greatest influence on both maximum stress and average strain among the remaining values, is selected as the parameter interval.

In summary, an initial optimization model for the torque sensor is established:

(8) $$\begin{array}{cc} & \max f\left(x\right)=\varepsilon_{t}\\ & s.t.g\left(x\right)=\sigma_{\max}-{\left[\sigma \right]}_{yield}\leq 0\\ & n=\frac{\sigma_{\max}}{{\left[\sigma \right]}_{yield}}\to 1\\ & x=[L2,L4,H]\\ & 8\;\mathrm{mm}\leq L2\leq 10\;\mathrm{mm}\\ & 3.75\;\mathrm{mm}\leq L4\leq 4.25\;\mathrm{mm}\\ & 6.5\;\mathrm{mm}\leq H\leq 7.5\;\mathrm{mm} \end{array}$$

**Figure 11:**
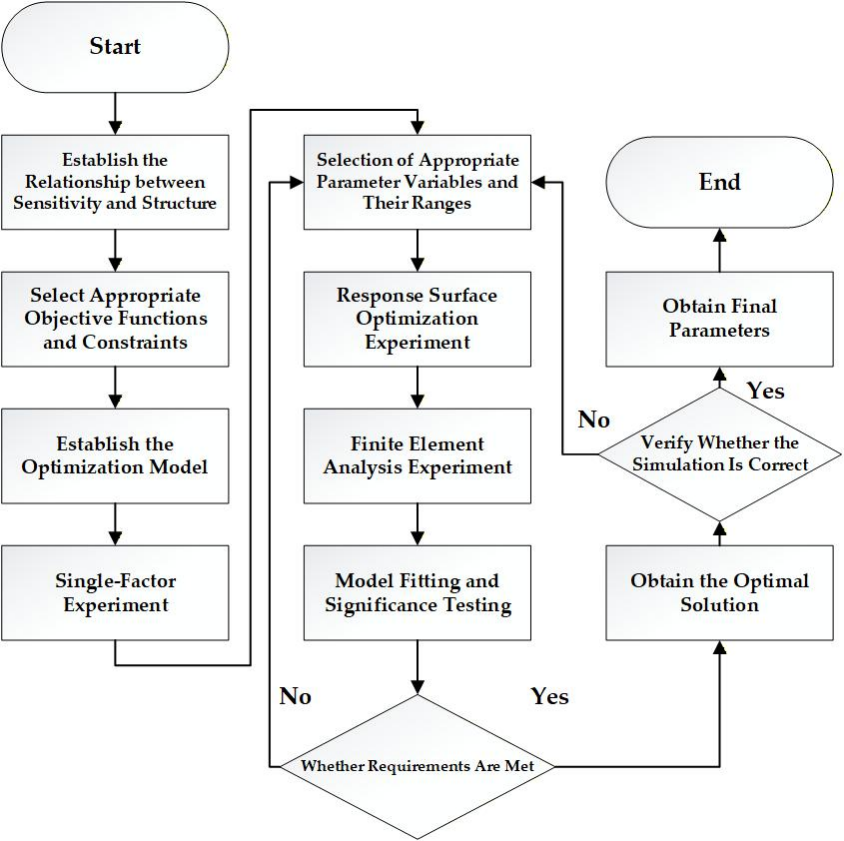
Optimization Workflow.

In summary, to identify the potential optimal solution of the model, it is first necessary to screen for factors that significantly influence the response outcome. For linear elastic materials such as 7075-T6, maximum stress exhibits a positive correlation with average strain. Given the constraint that the maximum stress must not exceed the yield strength, while aiming to maximize the average strain, the potential optimal solution invariably lies in the parameter space where the maximum strain approaches the yield strength. If the results from an initial response surface methodology (RSM) experiment fail to meet the expected performance evaluation criteria, the range of significant factors should be redefined following the procedure outlined in Figure 11, and a subsequent RSM optimization experiment should be conducted. This approach effectively reduces the number of ineffective RSM optimization trials and enhances overall optimization efficiency.

### 4.2. Optimization Strategy

Given that the optimization problem in this study involves multiple continuous design variables and that the responses can be obtained through simulation—exhibiting continuous and relatively smooth nonlinear characteristics within the research interval—the Response Surface Methodology (RSM) is employed for model optimization. Compared to intelligent optimization methods such as genetic algorithms, particle swarm optimization, and topological optimization techniques, RSM can establish a quadratic regression model with fewer experimental or computational iterations. This approach enables quantitative analysis of the main effects, quadratic effects, and interaction effects of each design variable, offering relatively high computational efficiency.

Furthermore, this study requires strict adherence to engineering constraints during the optimization process, categorizing it as a typical constrained parameter optimization problem. The constructed response surface model can effectively cover the design space without involving extreme operating conditions, thereby contributing to the safety and stability of experimental simulations. In contrast, while intelligent optimization algorithms possess strong global search capabilities, they typically require a large number of function evaluations, resulting in higher computational costs, and the obtained optimal solutions offer relatively limited interpretability in terms of engineering mechanisms. On the other hand, topological optimization may yield structures with features such as minute gaps or complex geometries, leading to difficulties in subsequent manufacturing. Additionally, it tends to be computationally less efficient and does not output parameterized data.

Therefore, after comprehensively considering computational efficiency, engineering feasibility, and model interpretability, this paper selects RSM for systematic optimization analysis.

There are several experimental design approaches within response surface methodology (RSM). Among them, the Box–Behnken Design (BBD) is particularly suitable for studies involving three to five factors, as it allows the investigation of nonlinear relationships and provides efficient analysis of two-factor interactions. Therefore, in this study, the BBD approach is employed to conduct a three-factor, three-level response surface experiment. The overall experimental workflow is illustrated in Figure 11.

### 4.3. Optimization Results

**Figure 12:**
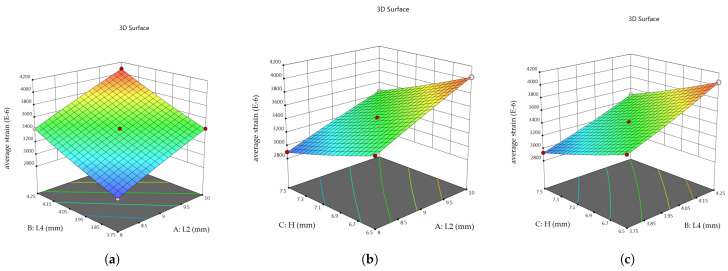
Response surface of average strain versus variables: (**a**) *L*2 and *L*4. (**b**) *L*2 and *H*. (**c**) *L*4 and *H*.

**Figure 13:**
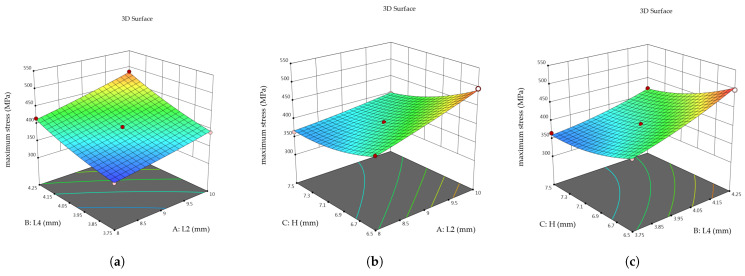
Response surface of maximum stress versus variables: (**a**) *L*2 and *L*4. (**b**) *L*2 and *H*. (**c**) *L*4 and *H*.

This study utilized Design-Expert software to conduct response surface fitting, where the selected parameters and their corresponding simulation outcomes were processed for analysis. The response surfaces illustrating the relationships between the variables and the objective functions are shown in Figure 12 and Figure 13.

By comparing models including linear, two-factor interaction, quadratic, and cubic equations, and evaluating their significance and lack-of-fit tests, the quadratic model was found to be the most appropriate. It provides the best representation of the relationships between the parameters and the response surface. The regression coefficients of the quadratic equation were obtained using the least-squares method, with non-significant terms omitted.

Accordingly, the average strain at the strain gauge location f(x) and the maximum stress of the elastic body g(x) are expressed as:

(9) $$\begin{array}{cc} & f\left(x\right)=3434.00+314.75\times L2+306.00\times L4-258.50\times H+16.00\times L2\times L4\\ & 29.50-\times L2\times H-20.00\times L4\times H-11.00\times L2^{2}+24.00\times L4^{2}+18.00\times H^{2} \end{array}$$

(10) $$\begin{array}{cc} & g\left(x\right)=399+30.00\times L2+39.38\times L4-32.13\times H+5.75\times L2\times L4-13.25\times \\ & L2\times H-10.00\times L4\times H+2.50\times L2^{2}+7.75\times L4^{2}+15.25\times H^{2} \end{array}$$

It can be observed that L2, L4, and H exert a significant influence on both the average strain f(x) and the maximum stress g(x) of the elastic body. Notably, H has a negative effect on the objective function, such that a smaller H corresponds to a higher value of the target strain.

Variance analysis of the objective function model yielded *p* < 0.0001 and R^2^ > 0.9, with the adjusted values closely matching the predicted values and a signal-to-noise ratio well above 4, indicating that the model is reliable. Finite element analysis of the optimized parameter model confirmed that the simulation results at the optimal solution were consistent with the optimization outcomes. The maximum stress did not exceed the material’s yield strength, ensuring no plastic deformation occurs. The ratio of the maximum stress to the material’s yield strength represents the material utilization rate. A comparison of the optimization results is presented in Table 5.

The final results indicate that, after optimization, the sensor’s material utilization rate increased by 13.1%, and its sensitivity improved by 11.9%.

## 5. Finite Element Analysis and Comparison

After determining the final optimized dimensions, an analysis was conducted to verify whether the sensor could withstand 250% overload capacity. In the finite element model, the inner flange of the elastic body was defined as a fixed constraint, while a torque load of 75 N·m was applied along the y−axis to the outer flange. The target response of this study is the average strain at the strain gauge attachment area on the elastic body; therefore, mesh convergence analysis was conducted using this average strain as the evaluation metric. By progressively refining the local mesh, convergence was considered achieved when the relative change in the average strain within this region fell below 1%. The final mesh scheme was selected to balance computational accuracy and efficiency for subsequent analysis.

**Figure 14:**
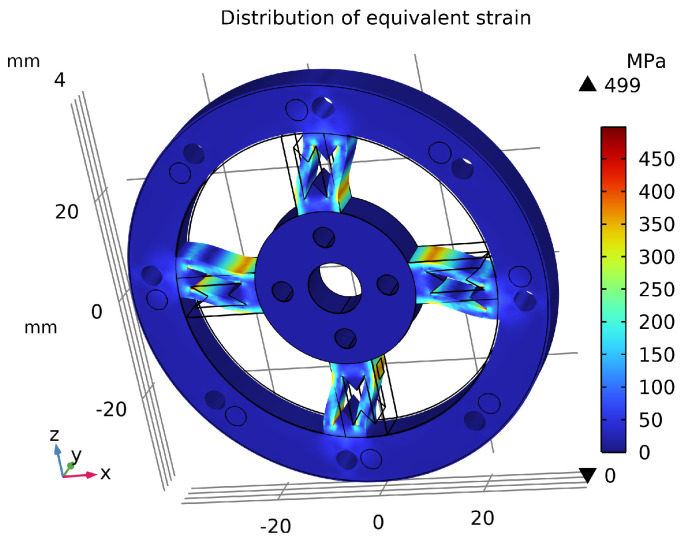
Equivalent stress distribution of the sensor.

**Figure 15:**
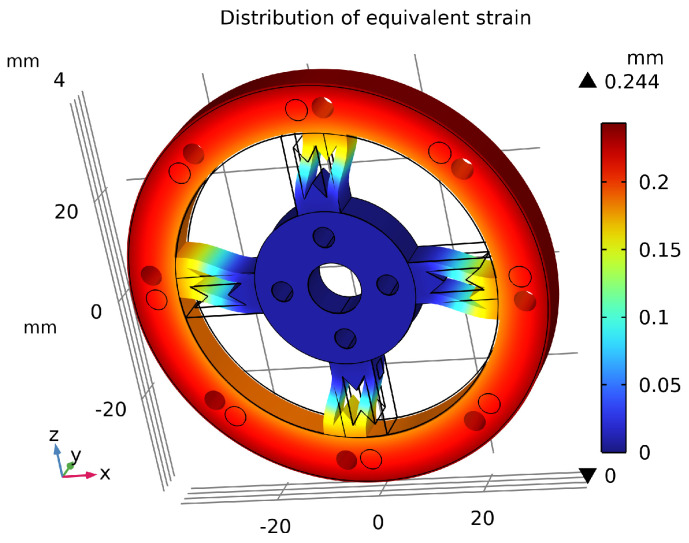
Displacement distribution of the sensor.

**Figure 16:**
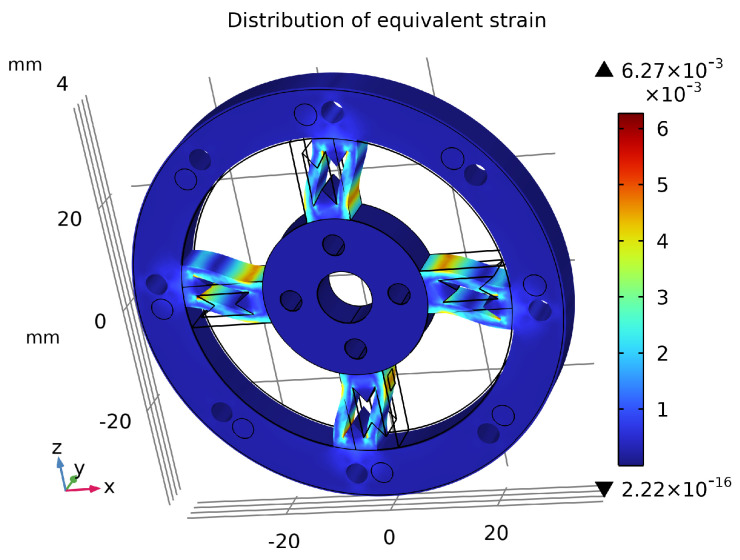
Equivalent strain distribution of the sensor.

**Figure 17:**
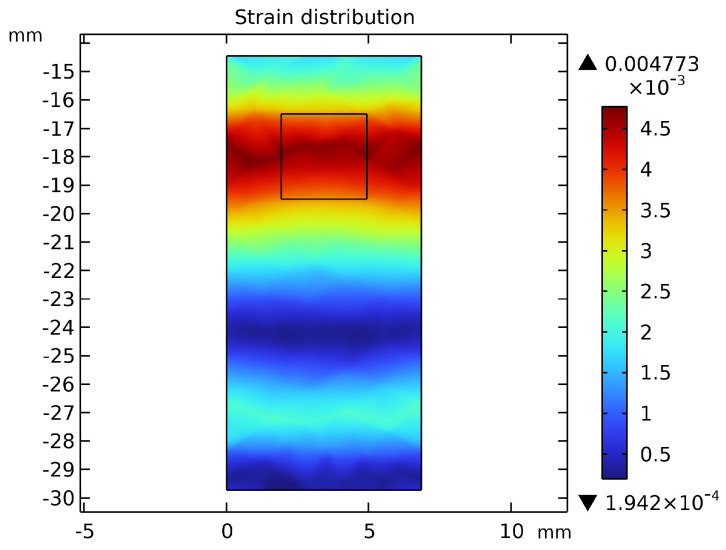
Strain distribution on sensor spokes.

As shown in Figure 14, the sensor’s maximum average equivalent stress reached 499 MPa, which does not exceed the material’s initial yield stress of 503 MPa, indicating no plastic deformation occurs. The maximum displacement under the applied torque was only 0.244 mm, as shown in Figure 15, which has negligible impact on the output signal transmission. Figure 16 illustrates the overall equivalent strain distribution of the elastic element under the 75 N·m torque; the strain distribution is uniform and well-defined. Figure 17 illustrates the strain distribution on the sensor spokes. Evidently, compared to the previously discussed non-apertured sensor, the hourglass-apertured sensor demonstrates a more uniform strain distribution concentrated predominantly in the central region of the spokes. This characteristic significantly facilitates subsequent strain measurement.

The hourglass-hole spoke-type sensor ultimately exhibited superior performance compared to the dumbbell-hole design in terms of the average strain at the strain gauge attachment points, maximum stress of the elastic element, material utilization, and sensitivity. The comparative results are summarized in Table 6.

## 6. Results

Based on the demand for high-precision torque sensing in robotic manipulators operating in complex environments, this study designed a high-sensitivity hourglass-hole spoke-type joint torque sensor through material performance comparison and structural selection. Furthermore, a comprehensive systematic response surface optimization methodology was proposed. Prior to response surface methodology (RSM) optimization, single-factor experiments for structural optimization and explanatory notes on parameter range selection were incorporated. This approach effectively screened for factors significantly influencing the target response and determined their reasonable experimental ranges, thereby enhancing both the efficiency and rigor of the optimization process. The optimized sensor exhibits a 13.1% improvement in material utilization and a 11.9% increase in sensitivity. Under an overload torque of 75 N·m, the maximum stress of the sensor remains below the yield strength, ensuring normal functionality even at 250% of the rated load. With an elastomer thickness of only 6.85 mm, the sensor achieves lightweight performance and facilitates integration into joint assemblies. Compared to a solid spoked sensor, the hourglass-hole design demonstrates more uniform strain distribution concentrated in the middle region of the spokes, simplifying strain gauge attachment and measurement. The sensitivity of the proposed sensor is 0.558 mV/N·m, which is approximately 19.2% higher than that of the dumbbell-hole type, while material utilization is improved by 3.1%.

## 7. Discussion

Although certain performance improvements have been achieved, it must be acknowledged that this study has certain limitations.

Firstly, the simulation model did not account for the influence of non-axial loads that may exist in practical applications, which could introduce interference in measurement accuracy. Therefore, simulation verification experiments on the effects of non-axial loads have been added. During the static calibration experiment, the gravitational force of the calibration arm may introduce lateral pressure interference on the sensor. Therefore, an additional validation experiment on lateral load interference was conducted to ensure the reliability of the calibration process. The calibration arm has a volume of approximately 64 cm^3^. To ensure higher stiffness than the elastic body, 45-gauge steel was selected as the material, resulting in a mass of 502.4 g for the calibration arm. The force-bearing area of the elastic body is 2792 mm^2^, leading to a lateral load pressure of 1852 Pa on the side of the elastic body. With the inner flange of the elastic body fixed as a constraint and a maximum rated torque load of 30 N·m applied along the y-axis to the outer flange, the average strain in the strain gauge attachment area was simulated under two conditions: with and without the pressure load. Without the pressure load, the average strain was $1673.6\times 10^{-6}$, whereas with the pressure load, it was $1673.4\times 10^{-6}$. The resulting non-axial load interference error caused by the gravitational effect of the calibration arm was only 0.012%, indicating that the sensor possesses excellent resistance to eccentric loading.

Second, all performance data are derived from simulations and require validation through physical experiments. To address these limitations, a clear experimental plan has been outlined for future work.

The first step involves prototype fabrication and signal conditioning circuit design. The sensor’s elastic body is manufactured with high precision, with critical dimensional tolerances maintained within ±0.01 mm. Strain gauge selection, bonding position determination, and surface preparation are performed. Precise strain gauge bonding and quality control are achieved using microscope-assisted alignment. A full-bridge configuration is adopted to maximize the output signal and provide temperature self-compensation. An amplification and filtering circuit is designed to effectively capture the weak signal, while a boost circuit and a voltage regulation circuit are incorporated to supply power to the bridge and the main control circuit, respectively. The amplified analog signal is digitized by an ADC and processed in real-time by a microcontroller. Ultimately, the processed torque data is transmitted to a host computer to ensure the accuracy and stability of the acquired data.

Subsequently, calibration experiments are designed, and a static calibration test bench is constructed. The sensor’s linearity, repeatability, hysteresis, and sensitivity coefficient are systematically measured, and the results are cross-validated against the simulation data.

## 8. Conclusions

This study designs and optimizes an hourglass-hole spoke-type torque sensor structure for torque sensing in robotic manipulator joints. The results demonstrate that the proposed design exhibits favorable performance in terms of sensitivity, material utilization, and structural compactness. Furthermore, the introduced systematic response surface optimization method proves applicable to the structural optimization of linear elastic materials, providing a feasible solution for achieving reliable torque sensing in robots operating in complex environments.

## Figures and Tables

**Table 1:** Sensor Design Specifications.

Parameter	Value
Dimensions (mm)	Ø78×Ø10×H7
Capacity (N·m)	30
Overload (%)	250
Minimum resolution (N·m)	0.1
Sensitivity (mV/N·m)	>0.468

**Table 2:** Detailed dimensional values.

Parameter	*D*1	*D*2	*D*3	*D*4	*L*1	*L*2	*L*3	*L*4
Value (mm)	78	60	30	10	22.5	10	3	4

**Table 3:** Comparative Analysis of Material Properties and Mechanical Simulation Results.

Property	7075-T6	6061-T6	2A12-T4	17-4PH	Ti-6Al-4V	42CrMo
Poisson’s Ratio	0.33	0.33	0.34	0.28	0.36	0.28
Density (kg/m^2^)	2.81	2.7	2.77	7.8	4.51	7.85
Young’s Modulus (GPa)	71.7	68.9	68	200	110	210
Yield Strength (MPa)	503	276	325	1000	930	930
Machinability	Easy	Easy	Easy	Moderate	Difficult	Moderate
Max Stress (MPa)	441	346	378	549	430	459
Average Strain (10^−6^)	3740	4026	3970	1298	2485	1236

**Table 4:** Selected Performance Comparison.

Structure Type	Solid Spoke	Dumbbell Hole	Hourglass Hole
Maximum Stress (MPa)	270	324	441
Material Utilization (%)	53.7	64.4	87.7
Average Strain (10^−6^)	1044	3000	3740
Sensitivity (mV/N·m)	0.139	0.400	0.499

**Table 5:** Optimized Parameter Results.

Name	*L*2 (mm)	*L*4 (mm)	*H* (mm)	g(x) (MPa)	f(x) $\left(10^{-6}\right)$
Before Optimization	10.00	4.00	7.00	441	3740
After Optimization	10.00	4.25	6.85	499	4184

**Table 6:** Comparison of Sensors with Different Hole Designs.

Hole Type	Dumbbell Hole	Hourglass Hole
Maximum Stress (MPa)	483.2	499
Material Utilization (%)	96.1	99.2
Average Strain (10^−6^)	3510	4184
Sensitivity (mV/N·m)	0.468	0.558

## Data Availability

The original contributions presented in this study are included in the article. Further inquiries can be directed to the corresponding author.
